# Free and Forced Vibration Analyses of Functionally Graded Graphene-Nanoplatelet-Reinforced Beams Based on the Finite Element Method

**DOI:** 10.3390/ma15176135

**Published:** 2022-09-04

**Authors:** Yuanxiu Zhang, Jingmei Teng, Jun Huang, Kun Zhou, Lixin Huang

**Affiliations:** 1 Key Laboratory of Disaster Prevention and Structural Safety of Ministry of Education, Guangxi University, Nanning 530004, China; 2 School of Civil Engineering and Architecture, Guangxi University, Nanning 530004, China; 3 College of Architecture and Civil Engineering, Nanning University, Nanning 530200, China; 4 Guangxi Key Laboratory of Disaster Prevention and Engineering Safety, Guangxi University, Nanning 530004, China

**Keywords:** functionally graded material, graphene-nanoplatelet-reinforced composite, finite element method, free vibration, transient response, Euler–Bernoulli beam theory

## Abstract

The finite element method (FEM) is used to investigate the free and forced vibration characteristics of functionally graded graphene-nanoplatelet-reinforced composite (FG-GPLRC) beams. The weight fraction of graphene nanoplatelets (GPLs) is assumed to vary continuously along the beam thickness according to a linear, parabolic, or uniform pattern. For the FG-GPLRC beam, the modified Halpin–Tsai micromechanics model is used to calculate the effective Young’s modulus, and the rule of mixture is used to determine the effective Poisson’s ratio and mass density. Based on the principle of virtual work under the assumptions of the Euler–Bernoulli beam theory, finite element formulations are derived to analyze the free and forced vibration characteristics of FG-GPLRC beams. A two-node beam element with six degrees of freedom is adopted to discretize the beam, and the corresponding stiffness matrix and mass matrix containing information on the variation of material properties can be derived. On this basis, the natural frequencies and the response amplitudes under external forces are calculated by the FEM. The performance of the proposed FEM is assessed, with some numerical results obtained by layering method and available in published literature. The comparison results show that the proposed FEM is capable of analyzing an FG-GPLRC beam. A detailed parametric investigation is carried out to study the effects of GPL weight fraction, distribution pattern, and dimensions on the free and forced vibration responses of the beam. Numerical results show that the above-mentioned effects play an important role with respect to the vibration behaviors of the beam.

## 1. Introduction

Graphene is a single two-dimensional honeycomb lattice structure composed of carbon atoms and the basic constituent of other graphite materials with varied dimensions [1]. Lee et al. [2] first measured the elastic properties and internal fracture strength of monolayer graphene by the nanoindentation method under atomic force microscopy (AFM). They found that graphene has a Young’s modulus of 1 ± 0.1 TPA and internal strength of 130 ± 10 GPa, which are superior to those of other materials. Graphene has been used by many researchers as a reinforcing material to improve the comprehensive properties of nanocomposites due to its thin thickness, strength, and thermal conductivity [3]. Rafiee et al. [4] carried out experimental studies on different types of epoxy nanocomposites and found that after 0.1% graphene nanoplatelets (GPLs) were added, the Young’s modulus of GPLs/epoxy resin composites increased by 31%, and the tensile strength increased by 40% compared with that of the original epoxy resin matrix composites. In contrast, the tensile strength of single-walled carbon nanotubes/epoxy resin and multiwalled carbon nanotubes/epoxy resin composites is only 11% and 14% higher than that of the original epoxy resin matrix, respectively, which indicates that graphene can significantly improve the mechanical properties of nanocomposites and is superior to carbon nanotubes. Mohammad et al. [4] experimentally investigated the mechanical properties of graphene/epoxy composites and found that the elastic modulus, tensile strength, and toughness of graphene/epoxy composites were improved by 31%, 40%, and 53%, respectively, with superior enhancements relative to those of fillers such as carbon nanotubes. Fang et al. [5] investigated graphene/epoxy nanocomposites by adding a 0.6% mass fraction of amino-modified graphene to the epoxy resin matrix, and the results showed that the fracture toughness and flexural strength of the composites were improved by 93.8% and 91.5%, respectively. Park et al. [6] prepared graphene-based polyimide (PI) nanocomposites using an effective method, and the results showed that the tensile modulus and strength increased by 170% and 64%, respectively, compared with pure polyimide. Zhao et al. [7] studied the mechanical properties of GPLs/polyvinyl alcohol (PVA) nanocomposites and found that when 1.8% volume graphene was added, the tensile strength of the graphene/PVA composite increased by 150%, and the Young’s modulus increased by 10 times. Liang et al. [8] prepared nanocomposites of polyvinyl alcohol (PVA) and graphene oxide (GO) using an aqueous solution treatment method and found that there was efficient load transfer between the nanofiller graphene and the matrix polyvinyl alcohol, and the tensile strength and Young’s modulus of the composites were improved by 76% and 62%, respectively, with only 0.7% mass fraction of graphene oxide added. Katsuyuki et al. [9] used a continuous, scalable SSSP process in which well-dispersed, unmodified graphite/polypropylene (PP) composites could be obtained, with a 100% increase in Young’s modulus and a 60% increase in yield strength of the composites relative to pure polypropylene (PP).

Functionally graded (FG) material is a type of non-homogeneous composite material first proposed by Bever et al. [10,11] in 1972, the components and properties of which change continuously in the direction of thickness or length to form a spatial gradient [12]. Yang and his team [13,14,15,16,17,18,19,20,21] first introduced the concept of FG into graphene composites in 2017, proposing the idea of graphene-enhanced functionally graded materials, in which GPLs are randomly and uniformly distributed in each layer and their weight fraction varies layer by layer along the thickness direction. In order to disperse more GPLs in the regions where they are most needed, they proposed functionally graded graphene-nanoplatelet-reinforced composite (FG-GPLRC) beam, arch, plate, and shell structures and investigated their static bending, free vibration, and dynamic response. A two-variable sinusoidal shear deformation theory (SSDT) and a nonlocal elasticity theory were used by Arefi et al. [22] to analyze the free vibration behavior of FG-GPLRC plates. They discussed the effects of factors such as GPLs parameters, plate parameters, and the parameters related to the Pasternak foundation and geometry on the free vibration response of the plates. In a subsequent study [23], used first-order shear deformation theory (FSDT) and non-local elastic theory to study the bending response parameters of GPL-reinforced FG polymer composite curved beams based on a Pasternak foundation. They discussed the effects of the weight fraction and geometrical features of GPLs on the kinematic and static behavior of the beams. Based on first-order shear deformation plate theory, Song et al. [24] investigated the effects of GPL distribution pattern, weight fraction, geometry and size, and total number of layers on the dynamic characteristics of functionally graded multilayered graphene nanoplate/polymer composite plates. Navier’s method was used to obtain the analytical solution for the governing partial differential equations. Baghbadorani et al. [25] investigated the free vibrations of graphene-platelet-reinforced composite (GPLRC) cylindrical shells based on first-order shear deformation theory. Using the appropriate technique of Navier solution, an analytical solution was provided to obtain the natural frequencies, as well as the number of modes. Niu et al. [26] investigated the free vibration characteristics of rotating pretwisted FG-GPLRC cylindrical shell plates under cantilever conditions using the Chebyshev–Ritz method and discussed the effects of factors such as GPL parameters, pretorsion angle, and rotational speed on the natural frequencies. Based on the third-order shear deformation theory, Li et al. [27] investigated the primary and secondary resonances of FG-GPLRC beams. They examined the effects of GPL parameters on the nonlinear response of the primary, secondary, and combination resonances and found that the addition of a very low-weight fraction of GPLs significantly reduced the resonances of the beams. Based on Hamilton’s principle and the nonlinear von Karman strain–displacement relationship, Wang et al. [28] studied the nonlinear transient dynamic response of FG-GPLRC double-curved shallow shells under time-varying blast loads. According to their reported results, the nonlinear transient response of the shell is considerably affected by many factors, such as the temperature field, GPL parameters, shell parameters, etc. Many achievements have been made with respect to analysis of the mechanical performance of FG-GPLRC structures, but few studies have been reported on FG-GPLRC structures using the finite element method (FEM).

As a powerful numerical method, the FEM is flexible in terms of application to complex geometric configurations and various physical problems. The advantages of the FEM are well-suited for analysis of FG-GPLRC structures. In the present study, the free and forced vibration responses of FG-GPLRC beams were investigated using the FEM. We assumed that the weight fraction of GPLs varies continuously along the beam thickness according to a linear, parabolic, or uniform pattern. The effective Young’s modulus was calculated according to the modified Halpin–Tsai micromechanics model, whereas the effective Poisson’s ratio and mass density were determined by the rule of mixture. Finite element formulations for the analysis of the FG-GPLRC beams were derived based on the principle of virtual work under the assumptions of the Euler–Bernoulli beam theory. Thus, the corresponding stiffness matrix and mass matrix containing information on the variation of material properties can be derived. The proposed finite element model was verified by ABAQUS based on the layering method and previously published results, confirming agreement. Finally, the effects of the GPL weight fraction, distribution pattern, and dimensions on the free and forced vibration responses of the beam were investigated.

## 2. Functionally Graded Graphene-Nanoplatelet-Reinforced Beam

**Figure 1:**
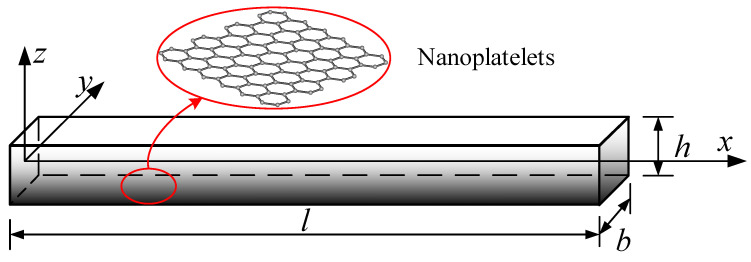
A functionally graded beam reinforced with graphene platelets.

**Figure 2:**
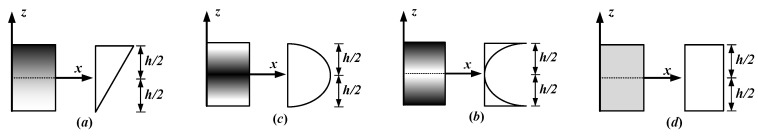
Four distribution patterns of GPLs along the thickness (**a**) LDP (**b**) PDPRS (**c**) PDPRM (**d**) UDP.

Figure 1 represents an FG beam (length, l; width, b; thickness, h) in which GPLs are distributed in the matrix according to a certain law, with the weight fraction of GPLs (WGPL) changing continuously along the thickness of the beam. Four distribution patterns of GPLs were considered, as shown in Figure 2 [14,15].

(a) Linear distribution pattern (LDP): The weight fraction of GPLs changes from the maximum value on the top surface to the minimum value on the bottom surface, along with the thickness, i.e.,

(1) $$W_{GPL}(z)=\lambda_{1}(\frac{1}{2}+\frac{z}{h})\;W_{GPL}^{0}$$

(b) Parabolic distribution pattern with rich GPLs in the surface (PDPRS): The weight fraction of GPLs is the highest at the top and bottom, lowest in the middle plane, and symmetric about the neutral axis, i.e.,

(2) $$W_{GPL}(z)=\frac{4}{h^{2}}\lambda_{2}z^{2}W_{GPL}^{0}$$

(c) Parabolic distribution pattern with rich GPLs in the middle plane (PDPRM): The weight fraction of GPLs is the lowest at the top and bottom, highest in the middle plane, and symmetric about the neutral axis, i.e.,

(3) $$W_{GPL}(z)=(1-\frac{4z^{2}}{h^{2}})\lambda_{3}W_{GPL}^{0}$$

(d) Uniform distribution pattern (UDP): The weight fraction of GPLs is evenly distributed in the beam, forming an isotropic, homogeneous beam, i.e.,
  (4) $$W_{GPL}(z)=\lambda_{4}W_{GPL}^{0}$$
  where $\lambda_{i}\;\left(i=1,\;2,\;3,\;4\right)$ is a gradient indicator controlling the distribution of the weight fraction of GPLs along the thickness direction; and $W_{GPL}^{0}$ is a specific weight fraction, which is taken as $W_{GPL}^{0}=1\%$ in this paper.

**Figure 3:**
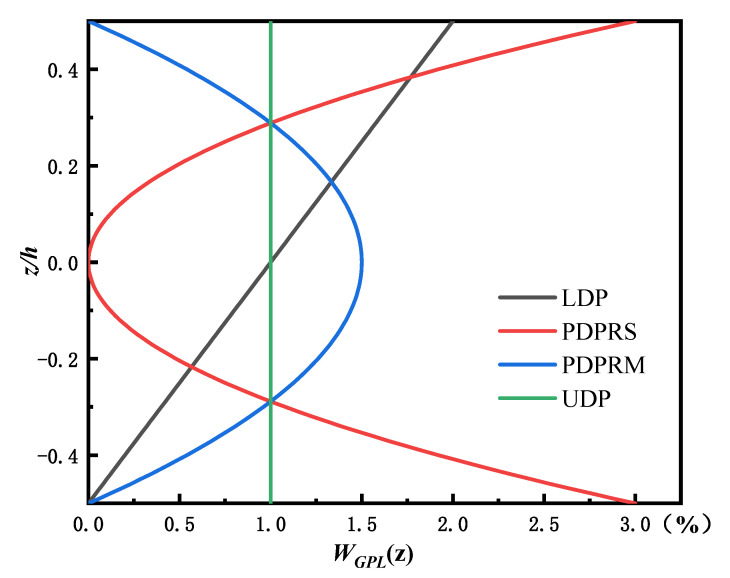
Distribution of graphene weight fraction along beam thickness.

$W_{GPL}^{t}$ represents the total weight fraction of GPLs in the whole beam and can be determined by integrating $W_{GPL}(z)$ along the thickness, i.e., $W_{GPL}^{t}=\int_{-h/2}^{h/2}W_{GPL}(z)\;dz$. After integrating Equations (1)–(4), the relationship between $W_{GPL}^{t}$ and $\lambda_{i}$ can be obtained, as listed in Table 1. The variation in GPL weight fraction along the thickness in the four distribution patterns is shown in Figure 3 when the overall weight fraction ($W_{GPL}^{t}$) is 1%.

The effective Young’s modulus ($E_{C}$) of graphene-reinforced composites can be calculated according to the modified Halpin–Tsai micromechanics model [13], i.e.,

(5) $$E_{C}=\frac{3}{8}\times \frac{1+\xi_{L}\eta_{L}V_{GPL}}{1-\eta_{L}V_{GPL}}\times E_{M}+\frac{5}{8}\times \frac{1+\xi_{w}\eta_{w}V_{GPL}}{1-\eta_{w}V_{GPL}}\times E_{M}$$

in which:

(6) $$\eta_{L}=\frac{(E_{GPL}/E_{M})-1}{(E_{GPL}/E_{M})+\xi_{L}}$$

(7) $$\eta_{w}=\frac{(E_{GPL}/E_{M})-1}{(E_{GPL}/E_{M})+\xi_{w}}$$

(8) $$\xi_{L}=\frac{2l_{GPL}}{t_{GPL}}$$

(9) $$\xi_{w}=\frac{2b_{GPL}}{t_{GPL}}$$

where $E_{M}$ and $E_{GPL}$ are the Young’s moduli of the polymer matrix and GPLs, respectively; $V_{GPL}$ is the volume fraction of GPLs; and *l_GPL_*, *b_GPL_*, and *t_GPL_* denote the length, width, and thickness of GPLs, respectively.

The effective Poisson’s ratio ($\nu_{C}$) and mass density ($\rho_{C}$) of graphene-reinforced composites are determined according to the rule of mixture [13], i.e.,

(10) $$\nu_{C}=\nu_{GPL}V_{GPL}+\nu_{M}(1-V_{GPL})$$

(11) $$\rho_{C}=\rho_{GPL}V_{GPL}+\rho_{M}(1-V_{GPL})$$

in which:

(12) $$V_{GPL}=\frac{W_{GPL}}{W_{GPL}+\frac{\rho_{GPL}}{\rho_{M}}(1-W_{GPL})}$$

where $\nu_{GPL}$, $\rho_{GPL}$, and *W_GPL_* are the Poisson’s ratio, mass density, and weight fraction of GPLs, respectively; and $\nu_{M}$ and $\rho_{M}$ are the Poisson’s ratio and mass density of the matrix, respectively.

## 3. Governing Equations

Based on the Euler–Bernoulli beam theory, the axial and transverse displacements at any point in the beam can be expressed as [29]:

(13) $$u(x,z,t)=u_{0}(x,t)-z\frac{\partial w_{0}(x,t)}{\partial x}$$

(14) $$w(x,z,t)=w_{0}(x,t)$$

where $u_{0}$ and $w_{0}$ are the axial displacement and transverse displacement of any point on the neutral axis of the beam, respectively, and *t* represents time.

Then, the displacement vector, $\left\{\gamma_{s}\right\}$, can be expressed as:

(15) $$\left\{\gamma_{s}\right\}=\left\{\begin{array}{l} u(x,z,t)\\ w(x,z,t) \end{array}\right\}=\left[\begin{array}{ccc} 1 & 0 & z\\ 0 & 1 & 0 \end{array}\right]\left\{\begin{array}{l} u_{0}(x,t)\\ w_{0}(x,t)\\ \partial w_{0}(x,t)/\partial x \end{array}\right\}$$

Considering a small deformation, the displacement–strain relationship can be expressed as:

(16) $$\varepsilon_{xx}=\frac{\partial u(x,z,t)}{\partial x}=\frac{\partial u_{0}(x,t)}{\partial x}-z\frac{\partial^{2}w_{0}(x,t)}{\partial x^{2}}$$

where $\varepsilon_{xx}$ is the axial strain.

Based on the linear elastic theory, the relationship between stress and strain follows Hooke’s law, and the strain–stress constitutive equation can be expressed as:

(17) $$\sigma_{xx}=E(z)\varepsilon_{xx}=E(z)\left[\begin{array}{cc} 1 & -z \end{array}\right]\left\{\begin{array}{l} \frac{\partial u_{0}(x,t)}{\partial x}\\ \frac{\partial^{2}w_{0}(x,t)}{\partial x^{2}} \end{array}\right\}$$

where E(z) is the Young’s modulus, and $\sigma_{xx}$ is the axial stress.

## 4. Finite Element Formulations

### 4.1. Dynamic Equations

The equations that govern the dynamic response of a structure can be obtained based on the principle of virtual work [30,31]. For the FG-GPLRC beam, the virtual work equation becomes:

(18) $$\delta W_{S}+\delta W_{C}+\delta W_{I}=\delta W_{F}$$

where $W_{S}$ and $W_{C}$ are the sums of the work done by the stress and damping force, respectively; $W_{I}$ is the sum of the work done by the inertia force; and $W_{F}$ is the sum of the work done by the external load.

The work done by the stress on the virtual strain is:

(19) $$\delta W_{S}={∭}_{V}{\left[\sigma \right]}^{T}\left[\delta \varepsilon \right]dxdydz$$

Substituting Equations (16) and (17) into Equation (19) yields:

(20) $$\delta W_{S}=b\int_{0}^{L}\left\{\begin{array}{cc} \frac{\partial u_{0}(x,t)}{\partial x} & \frac{\partial^{2}w_{0}(x,t)}{\partial x^{2}} \end{array}\right\}\left[\int_{-h/2}^{h/2}\left[\begin{array}{cc} E(z) & -zE(z)\\ -zE(z) & z^{2}E(z) \end{array}\right]dz\right]\delta \left\{\begin{array}{l} \frac{\partial u_{0}(x,t)}{\partial x}\\ \frac{\partial^{2}w_{0}(x,t)}{\partial x^{2}} \end{array}\right\}dx$$

The work done by the damping forces on the virtual displacement is:

(21) $$\delta W_{C}=-{∭}_{V}c_{d}{\left\{{\dot{\gamma }}_{s}\right\}}^{T}\left\{\delta \gamma_{s}\right\}dxdydz$$

where $c_{d}$ is the material damping parameter.

Substituting Equation (15) into Equation (21) yields:

(22) $$\delta W_{C}=-bc_{d}\int_{0}^{L}\left\{\begin{array}{ccc} {\dot{u}}_{0}(x,t) & {\dot{w}}_{0}(x,t) & \frac{\partial {\dot{w}}_{0}(x,t)}{\partial x} \end{array}\right\}\left[\int_{-h/2}^{h/2}\left[\begin{array}{ccc} 1 & 0 & -z\\ 0 & 1 & 0\\ -z & 0 & z^{2} \end{array}\right]dz\right]\delta \left\{\begin{array}{l} u_{0}(x,t)\\ w_{0}(x,t)\\ \partial w_{0}(x,t)/\partial x \end{array}\right\}dx$$

The work done by the inertia force on the virtual displacement is:

(23) $$\delta W_{I}=-{∭}_{V}\rho {\left\{{\ddot{\gamma }}_{s}\right\}}^{T}\left\{\delta \gamma_{s}\right\}dxdydz$$

Substituting Equation (15) into Equation (23) yields:

(24) $$\delta W_{I}=-b\int_{0}^{L}\left\{\begin{array}{ccc} {\ddot{u}}_{0}(x,t) & {\ddot{w}}_{0}(x,t) & \frac{\partial {\ddot{w}}_{0}(x,t)}{\partial x} \end{array}\right\}\left[\int_{-h/2}^{h/2}\left[\begin{array}{ccc} \rho & 0 & -\rho z\\ 0 & \rho & 0\\ -\rho z & 0 & \rho z^{2} \end{array}\right]dz\right]\delta \left\{\begin{array}{l} u_{0}(x,t)\\ w_{0}(x,t)\\ \partial w_{0}(x,t)/\partial x \end{array}\right\}dx$$

The work done by the external force on the virtual displacement is:

(25) $${\delta W}_{F}=\int_{0}^{L}\left[\begin{array}{c} 0\\ fi\\ 0 \end{array}\right]\delta \left\{\begin{array}{l} u_{0}(x,t)\\ w_{0}(x,t)\\ \partial w_{0}(x,t)/\partial x \end{array}\right\}dx$$

### 4.2. Beam Element for FG Beam Reinforced with GPLs

**Figure 4:**
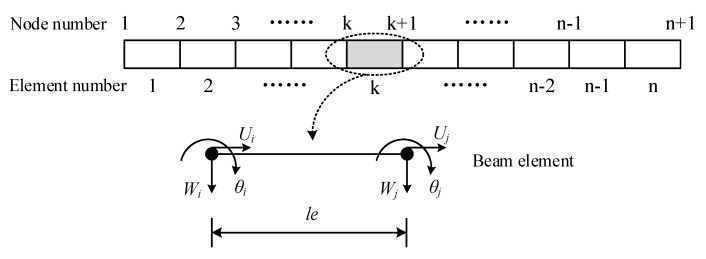
Beam discretization by two-node beam element with six degrees of freedom.

As shown in Figure 4, the FG-GPLRC beam is discretized by a two-node beam element with six degrees of freedom.

The components of element displacements at the mid plane of a beam can be expressed as [32]:

(26) $$u_{0}^{e}(x,t)=N_{3i-2}U_{i}(t)+N_{3j-2}U_{j}(t)$$

(27) $$w_{0}^{e}(x,t)=N_{3i-1}W_{i}(t)+N_{3i}\theta_{i}(t)+N_{3j-1}W_{j}(t)+N_{3j}\theta_{j}(t)$$

where $U_{i}$ and $W_{i}$ denote the axial and transverse displacements of node *i*; $\theta_{i}$ is the slope of node *i*; $U_{j}$ and $W_{j}$ denote the axial and transverse displacements of node *j*, respectively; $\theta_{j}$ is the slope of node *j*; $N_{3i-2}$ and $N_{3j-2}$ are Lagrange’s linear interpolation polynomials; and $N_{3i-1}$, $N_{3j-1}$, $N_{3i}$, and $N_{3j}$ are first-order Hermite’s interpolation polynomials, i.e.,

(28) $$N_{3i-2}=(1-\frac{x}{l_{e}})$$

(29) $$N_{3i-1}=\frac{1}{l_{e}{}^{3}}(l_{e}{}^{3}-3l_{e}x^{2}+2x^{3})$$

(30) $$N_{3i}=\frac{1}{l_{e}{}^{2}}(l_{e}{}^{2}x-2l_{e}x^{2}+x^{3})$$

(31) $$N_{3j-2}=\frac{x}{l_{e}}$$

(32) $$N_{3j-1}=\frac{1}{l_{e}{}^{3}}(3l_{e}x^{2}-2x^{3})$$

(33) $$N_{3j}=\frac{1}{l_{e}{}^{2}}(-l_{e}x^{2}+x^{3})$$

Because the virtual displacements are arbitrary, the combination of Equations (20), (22), (24), (25), and (26)–(33) yields:

(34) $$\left[M\right]\left\{{\ddot{r}}_{\left(t\right)}\right\}+\left[C\right]\left\{{\dot{r}}_{\left(t\right)}\right\}+\left[K\right]\left\{r_{\left(t\right)}\right\}=\left\{F_{\left(t\right)}\right\}$$

where [M] is the global mass matrix, [K] is the global stiffness matrix, [C] is the damping matrix, $\left\{F_{\left(t\right)}\right\}$ is the nodal load vector, and $\left\{r_{\left(t\right)}\right\}$ is the displacement vector.

The global stiffness matrix is expressed as:

(35) $$\left[K\right]=\sum_{n=1}^{numel}{\left[K\right]}^{e}$$

where *numel* denotes the total number of elements; and ${\left[K\right]}^{e}$ is the element stiffness matrix, which can be expressed as follows:

(36) $${\left[K\right]}^{e}=b\int_{0}^{l}{\left[B\right]}^{T}{\left[D\right]}_{E}\left[B\right]dx$$

in which:

(37) $$\left[B\right]=\left[\begin{array}{cccccc} \frac{\partial N_{3i-2}}{\partial x} & 0 & 0 & \frac{\partial N_{3j-2}}{\partial x} & 0 & 0\\ 0 & \frac{\partial^{2}N_{3i-1}}{\partial x^{2}} & \frac{\partial^{2}N_{3i}}{\partial x^{2}} & 0 & \frac{\partial^{2}N_{3j-1}}{\partial x^{2}} & \frac{\partial^{2}N_{3j}}{\partial x^{2}} \end{array}\right]$$

(38) $${\left[D\right]}_{E}=\int_{-h/2}^{h/2}\left[\begin{array}{cc} E(z) & -zE(z)\\ -zE(z) & z^{2}E(z) \end{array}\right]dz$$

Considering Equation (5), substituting Equations (37) and (38) into Equation (36) yields the integrand that contains functions of *x*. Thus, integration of the integrand in *x* over elemental length $l_{e}$ and the element stiffness matrix ${\left[K\right]}^{e}$ becomes:

(39) $${\left[K\right]}^{\left(e\right)}=\left[\begin{array}{cccccc} \frac{b}{l_{e}}E_{1} & 0 & -\frac{b}{l_{e}}E_{2} & -\frac{b}{l_{e}}E_{1} & 0 & \frac{b}{l_{e}}E_{2}\\ 0 & \frac{12b}{l_{e}^{3}}E_{3} & \frac{6b}{l_{e}^{2}}E_{3} & 0 & -\frac{12b}{l_{e}^{3}}E_{3} & \frac{6b}{l_{e}^{2}}E_{3}\\ -\frac{b}{l_{e}}E_{2} & \frac{6b}{l_{e}^{2}}E_{3} & \frac{4b}{l_{e}^{}}E_{3} & \frac{b}{l_{e}}E_{2} & -\frac{6b}{l_{e}^{2}}E_{3} & \frac{2b}{l_{e}^{}}E_{3}\\ -\frac{b}{l_{e}}E_{1} & 0 & \frac{b}{l_{e}}E_{2} & \frac{b}{l_{e}}E_{1} & 0 & -\frac{b}{l_{e}}E_{2}\\ 0 & -\frac{12b}{l_{e}^{3}}E_{3} & -\frac{6b}{l_{e}^{2}}E_{3} & 0 & \frac{12b}{l_{e}^{3}}E_{3} & -\frac{6b}{l_{e}^{2}}E_{3}\\ \frac{b}{l_{e}}E_{2} & \frac{6b}{l_{e}^{2}}E_{3} & \frac{2b}{l_{e}^{}}E_{3} & -\frac{b}{l_{e}}E_{2} & -\frac{6b}{l_{e}^{2}}E_{3} & \frac{4b}{l_{e}^{}}E_{3} \end{array}\right]$$

in which:

(40) $$E_{1}=\frac{3}{8}C_{1l}E_{M}+\frac{5}{8}C_{1w}E_{M}$$

(41) $$E_{2}=\frac{3}{8}C_{2l}E_{M}+\frac{5}{8}C_{2w}E_{M}$$

(42) $$E_{3}=\frac{3}{8}C_{3l}E_{M}+\frac{5}{8}C_{3w}E_{M}$$

where explicit forms of $C_{jl}$ and $C_{jw}$ (*j* = 1, 2, 3) are given in Appendix A.

The global mass matrix is expressed as:

(43) $$\left[M\right]=\sum_{n=1}^{numel}{\left[M\right]}^{e}$$

where ${\left[M\right]}^{e}$ is the element mass matrix, which can be expressed as:

(44) $${\left[M\right]}^{e}=b\int_{0}^{l}{\left[N\right]}^{T}{\left[D\right]}_{R}\left[N\right]dx$$

in which:

(45) $$\left[N\right]=\left[\begin{array}{cccccc} N_{3i-2} & 0 & 0 & N_{3j-2} & 0 & 0\\ 0 & N_{3i-1} & N_{3i} & 0 & N_{3j-1} & N_{3j}\\ 0 & \frac{\partial N_{3i-1}}{\partial x} & \frac{\partial N_{3i}}{\partial x} & 0 & \frac{\partial N_{3j-1}}{\partial x} & \frac{\partial N_{3j}}{\partial x} \end{array}\right]$$

(46) $${\left[D\right]}_{R}=\int_{-\frac{h}{2}}^{\frac{h}{2}}\left[\begin{array}{ccc} \rho & 0 & -\rho z\\ 0 & \rho & 0\\ -\rho z & 0 & \rho z^{2} \end{array}\right]dz$$

Similarly, considering Equation (11), substituting Equations (45) and (46) into Equation (44) yields the integrand that contains functions of *x*. Thus, integration of the integrand in *x* over elemental length $l_{e}$ and the element mass matrix ${\left[M\right]}^{e}$ becomes:

(47) $${\left[M\right]}^{\left(e\right)}=[\begin{array}{ccc} \frac{bl_{e}}{3}\rho_{1} & -\frac{b}{2}\rho_{2} & \frac{bl_{e}}{12}\rho_{2}\\ -\frac{b}{2}\rho_{2} & \frac{13bl_{e}}{35}\rho_{1}+\frac{6b}{5l_{e}}\rho_{3} & \frac{11bl_{e}^{2}}{210}\rho_{1}+\frac{b}{10}\rho_{3}\\ \frac{bl_{e}}{12}\rho_{2} & \frac{11bl_{e}^{2}}{210}\rho_{1}+\frac{b}{10}\rho_{3} & \frac{bl_{e}^{3}}{105}\rho_{1}+\frac{2bl_{e}}{15}\rho_{3}\\ \frac{bl_{e}}{6}\rho_{1} & -\frac{b}{2}\rho_{2} & -\frac{bl_{e}}{12}\rho_{2}\\ \frac{b}{2}\rho_{2} & \frac{9bl_{e}}{70}\rho_{1}-\frac{6b}{5l_{e}}\rho_{3} & \frac{13bl_{e}^{2}}{420}\rho_{1}-\frac{b}{10}\rho_{3}\\ -\frac{bl_{e}}{12}\rho_{2} & -\frac{13bl_{e}^{2}}{420}\rho_{1}+\frac{b}{10}\rho_{3} & -\frac{bl_{e}^{3}}{140}\rho_{1}-\frac{bl_{e}}{30}\rho_{3} \end{array}\;\begin{array}{ccc} \frac{bl_{e}}{6}\rho_{1} & \frac{b}{2}\rho_{2} & -\frac{bl_{e}}{12}\rho_{2}\\ -\frac{b}{2}\rho_{2} & \frac{9bl_{e}}{70}\rho_{1}-\frac{6b}{5l_{e}}\rho_{3} & -\frac{13bl_{e}^{2}}{420}\rho_{1}+\frac{b}{10}\rho_{3}\\ -\frac{bl_{e}}{12}\rho_{2} & \frac{13bl_{e}^{2}}{420}\rho_{1}-\frac{b}{10}\rho_{3} & -\frac{bl_{e}^{3}}{140}\rho_{1}-\frac{bl_{e}}{30}\rho_{3}\\ \frac{bl_{e}}{3}\rho_{1} & \frac{b}{2}\rho_{2} & \frac{bl_{e}}{12}\rho_{2}\\ \frac{b}{2}\rho_{2} & \frac{13bl_{e}}{35}\rho_{1}+\frac{6b}{5l_{e}}\rho_{3} & \frac{11bl_{e}^{2}}{210}\rho_{1}+\frac{b}{10}\rho_{3}\\ \frac{bl_{e}}{12}\rho_{2} & \frac{11bl_{e}^{2}}{210}\rho_{1}+\frac{b}{10}\rho_{3} & \frac{bl_{e}^{3}}{105}\rho_{1}+\frac{2bl_{e}}{15}\rho_{3} \end{array}]$$

in which:

(48) $$\rho_{1}=\rho_{M}h+\left(\rho_{GPL}-\rho_{M}\right)C_{1}$$

(49) $$\rho_{2}=\rho_{M}h+\left(\rho_{GPL}-\rho_{M}\right)C_{2}$$

(50) $$\rho_{3}=\rho_{M}h+\left(\rho_{GPL}-\rho_{M}\right)C_{3}$$

where explicit forms of $C_{j}$ (*j* = 1, 2, 3) are given in Appendix A.

### 4.3. Free Vibration Analysis

When the damping effect and external force are not considered, Equation (34) can be simplified as:

(51) $$\left[M\right]\left\{{\ddot{r}}_{\left(t\right)}\right\}+\left[K\right]\left\{r_{\left(t\right)}\right\}=0$$

This is the free vibration equation of the system, also known as the dynamic characteristic equation.

Suppose that:

(52) $$\left\{r_{\left(t\right)}\right\}=\left\{\phi_{n}\right\}\sin\omega_{n}(t-t_{0})$$

where $\left\{\phi_{n}\right\}$ is the mode shape vector for the *n*th-order mode of vibration, $\omega_{n}$ is the frequency of vibration of the $\phi_{n}$ vector, *t* is the time variable, and *t*_0_ is the time constant determined by the initial conditions.

Substituting Equation (52) into Equation (51) yields:

(53) $$\left[K\right]\left\{\phi_{n}\right\}-\omega_{n}{}^{2}\left[M\right]\left\{\phi_{n}\right\}=0$$

According to the theory of homogeneous equations, Equation (53) can be expressed as:

(54) $$\left[K\right]-\omega_{n}{}^{2}\left[M\right]=0$$

Thus, $\omega_{n}$ and $\left\{\phi_{n}\right\}$ can be obtained by solving Equations (54) and (53), respectively.

### 4.4. Transient Analysis

Equation (34) is the dynamic equation of the system. The modal superposition method [33,34] is adopted to analyze the modal transient response of the system. By projecting the physical model into the eigenmodal coordinate system, a set of mutually independent single-degree-of-freedom equations of motion can be obtained, i.e.,

(55) $$\ddot{q}+2\xi \dot{q}+\omega_{n}^{2}q=f_{t}=f_{t-\Delta t}+\frac{\Delta f}{\Delta t}\Delta t$$

The general form of the solution is:

(56) $$\left\{\begin{array}{c} q_{t+\Delta t}\\ {\dot{q}}_{t+\Delta t} \end{array}\right\}=\left[\begin{array}{cc} a_{11} & a_{12}\\ a_{21} & a_{22} \end{array}\right]\left\{\begin{array}{c} q_{t}\\ {\dot{q}}_{t} \end{array}\right\}+\left[\begin{array}{cc} b_{11} & b_{12}\\ b_{21} & b_{22} \end{array}\right]\left\{\begin{array}{c} f_{t}\\ {\dot{f}}_{t+\Delta t} \end{array}\right\}$$

At this point, the dynamic response under modal coordinates can be obtained, and the physical coordinate response of the system can be obtained through modal superposition, i.e.,:

(57) $$r_{\left(t\right)}=\sum_{n}\phi_{n}q_{n}$$

## 5. Numerical Results

The above framework offers the same procedures as those used in the standard FEM to analyze FG-GPLRC beams with elastic models and the procedures programmed with FORTRAN language through subroutine UEL of ABAQUS.

**Figure 5:**
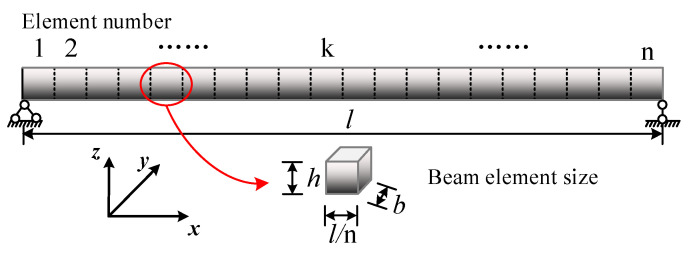
Finite element model of a simply supported FG-GPLRC beam.

Unless otherwise stated, the following conditions are used to investigate the effects of factors, such as the total weight fraction, distribution patterns, and size of GPLs, on the free vibration and transient response of the FG-GPLRC beam. Epoxy resin is chosen as the composite matrix, and GPLs are used as material reinforcement for this study. The material-related parameters are shown in Table 2 [35]. The FG beam length (*l* = 0.2 m) and thickness (*h*) vary with the length-to-slenderness ratio. The support conditions are selected to be simply supported at both ends, as shown in Figure 5. The following quantities can be defined as follows:

Dimensionless fundamental frequency:

$${\bar{\omega }}_{n}=\frac{\omega_{n}l^{2}}{h}\sqrt{\frac{\rho_{M}}{E_{M}}}$$

Dimensionless response vibration amplitude:

$$\bar{A}=100r_{\left(t\right)}\frac{E_{M}I}{F_{0}l^{4}}$$

where *F*_0_ is the maximum value of the dynamic load, and $I=\frac{bh^{3}}{12}$.

### 5.1. Convergence and Validation

The two-node beam element with six degrees of freedom is used to discretize the FG-GPLRC beam with a length-to-thickness ratio of 25 and a total GPLs weight fraction of 1%. The number of elements varies from 5 to 60 in order to investigate the convergence. Table 3 illustrates the calculated first-order dimensionless free vibration frequencies for four distribution patterns of GPLs. As shown in Table 3, the frequencies decrease continuously and converge to a certain value with an increase in the number of elements from 5 to 20, and the frequency values remain unchanged after the number of elements exceeds 20. According to the results, the number of elements has an impact on the calculation accuracy, and the calculated values correspond with the increase in the number of elements. Considering the calculation efficiency and accuracy, the number of elements is taken as 20 for all cases in the following calculation.

**Figure 6:**
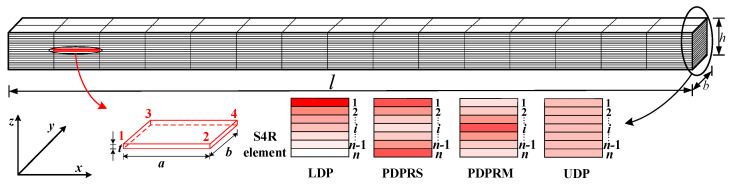
FEM mesh based on layering method.

Owing to the lack of suitable data available in the published literature for validation analysis, the solutions generated by finite element software ABAQUS for FG-GPLRC beams are chosen as the reference solutions to verify the proposed method. To solve the FG material inhomogeneity problem, a layering method [36,37,38] is used to divide the FG material into a certain number of homogeneous layers with the same material parameters in the same layer but with different material parameters in each layer. Figure 6 represents a schematic diagram of FEM mesh based on the layering method for FG-GPLRC beams. In the finite element model, the number of homogeneous layers is taken as 20, and the layers are discretized by the four-node S4R shell element with a size ratio of *a*: *b*: *t* = 25:10:1.

Both the proposed method based on a two-node beam element with six degrees of freedom and the ABAQUS solution based on a layering method are used to solve this problem. In addition, further verification results according to a numerical model are given in reference [35]. Under different GPL weight fractions, slenderness ratios, and GPL distribution patterns for a simply supported FG-GPLRC beam, the dimensionless fundamental frequencies obtained by the proposed method, ABAQUS, and Wang [35] are presented in Table 4. A comparison of the results shows that the solutions of the proposed method are in agreement with those of ABAQUS and Wang [35]. The maximum deviation of the presented solutions with those of Wang [35] is 0.576% (*l*/*h* = 25, PDPRS, $W_{GPL}^{t}=1\%$) and the maximum deviation of the presented solutions with those of ABAQUS is 0.920% (*l*/*h* = 20, PDPRS, $W_{GPL}^{t}=1\%$), which indicates that the proposed method highly accurate and is trustworthy. In addition, fewer elements are used to model the beam with the proposed method than the ABAQUS solution based on a laying method, indicating the efficiently of the proposed method.

The investigation of transient response is based on the modal superposition method, which first required determination of the free vibration frequency. Because no similar results have been published in the literature with respect to the transient response of the problem, in this study, we directly compare the amplitude of dimensionless transient response in the middle span of FG-GPLRC beams with the ABAQUS results. When $W_{GPL}^{t}=0.5\%$, the dynamic load applied in the span of the beam adopts the following form:

(58) $$F(t)=\{\begin{array}{cc} q & 0\leq t\leq 0.02s\\ 0 & t>0.02s \end{array}$$

**Figure 7:**
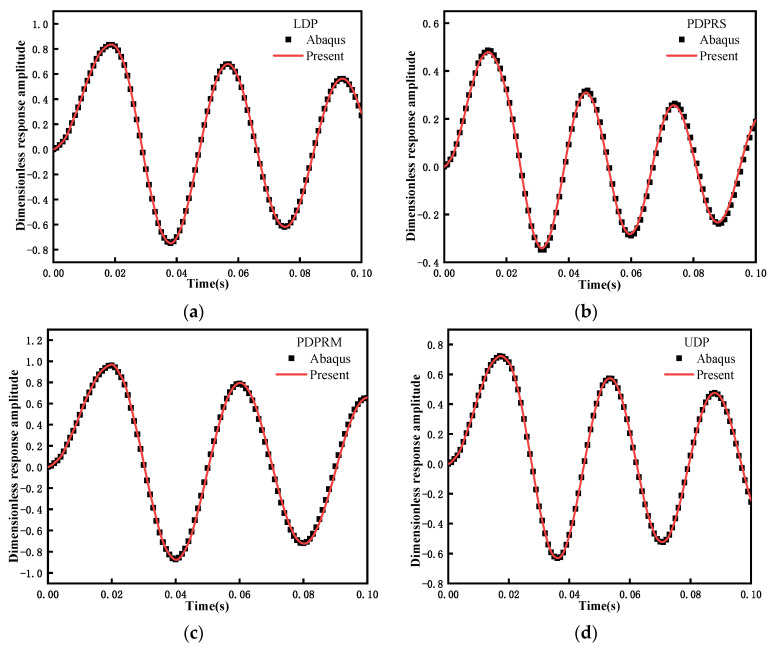
Comparisons of the transient response of the FG-GPLRC beam with four GPL distribution patterns using different methods: (**a**) LDP, (**b**) PDPRS, (**c**) PDPRM, and (**d**) UDP.

As shown in Figure 7, the results of the proposed method agree with the ABAQUS data under four distribution patterns of GPLs. Therefore, the reliability of the proposed method is further verified.

### 5.2. Free Vibration

**Figure 8:**
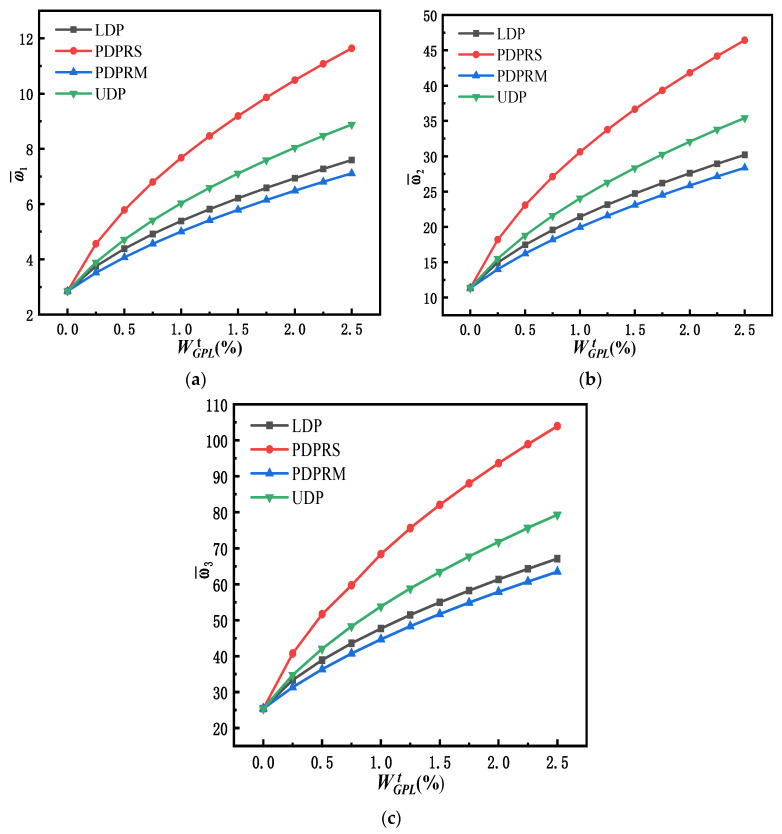
Variation of first three dimensionless natural frequencies with respect to the GPL weight fraction (*l/h* = 20). (**a**) The first dimensionless fundamental frequency; (**b**) the second dimensionless fundamental frequency; (**c**) the third dimensionless fundamental frequency.

The relationship between the first three-order dimensionless frequencies and the total weight fraction of GPLs is shown in Figure 8 for simply supported FG-GPLRC beams with four different GPL distribution patterns and a slenderness ratio of *l*/*h* = 20. The dimensionless frequency increases significantly after adding a small amount of graphene. In order to explore the effect of the weight fraction and distribution pattern of GPLs on the frequency, Table 5 lists the specific values of the first three dimensionless frequencies of FG-GPLRC beams with 0%, 0.5%, and 1% weight fractions of GPLs, respectively.

For the LDP of GPLs, the first three-order dimensionless frequencies increase by 54.006%, 53.815%, and 53.129% with the addition of a 0.5% weight fraction of GPLs and by 89.248%, 88.881%, and 87.740% with the addition of a 1.0% weight fraction of GPLs. Like the LDP of GPLs, PDPRS, PDPRM, and UDP are subject to a similar effect of the weight fraction of GPLs on the frequency. The addition of a small amount of graphene can greatly increase the frequency because graphene with a high Young’s modulus increases the stiffness of the beam. Under the same GPL distribution pattern, when the weight fraction of GPLs is the same, the growth rate of different order frequencies is almost the same, which indicates that there is no obvious difference in terms of the effect of the GPL weight fraction on different order frequencies.

Under the same GPL weight increment, such as a $W_{GPL}^{t}$ increase from 0.0% to 0.5%, the growth rate of different order frequencies is about 103% for the PDPRS of GPLs, 65% for the UDP of GPLs, 53% for the LDP of GPLs, and 43% for the PDPRM of GPLs. The distribution pattern of GPLs considerably affects the frequency of FG-GPLRC beams. When the addition of GPL weight fraction is the same, the PDPRS of GPLs has the greatest influence on the frequency, whereas the PDPRM of GPLs has the least influence on the frequency. This means that distributing more GPLs on the upper and lower surfaces of the beam is the most effective method to increase the flexural stiffness and the frequency of the beam.

**Figure 9:**
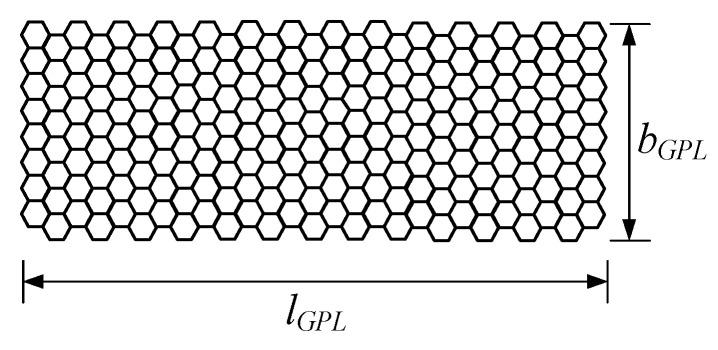
Schematic diagram of graphene nanosheets.

In order to study the effect of the geometric shape and size of GPLs on the dynamic performance of beams, three sizes of GPLs are considered, as shown in Figure 9. The free vibration characteristics of simply supported FG-GPLRC with a slenderness ratio of *l*/*h* = 20 are studied by adding GPLs of varying sizes, with the *GPL* weight fraction kept constant ($W_{GPL}^{t}$ = 1%). In this example, the length of GPLs (*l_GPL_*) remains unchanged, and the aspect ratio *l_GPL_*/*b_GPL_* = 1 corresponds to square GPLs, whereas the aspect ratios *l_GPL_*/*b_GPL_* = 2 and *l_GPL_*/*b_GPL_* = 3 correspond to two rectangular GPLs of different sizes. A higher length–thickness ratio (*l_GPL_*/*t*_GPL_) indicates that the GPLs are thinner and consist of fewer monolayers of graphene.

**Figure 10:**
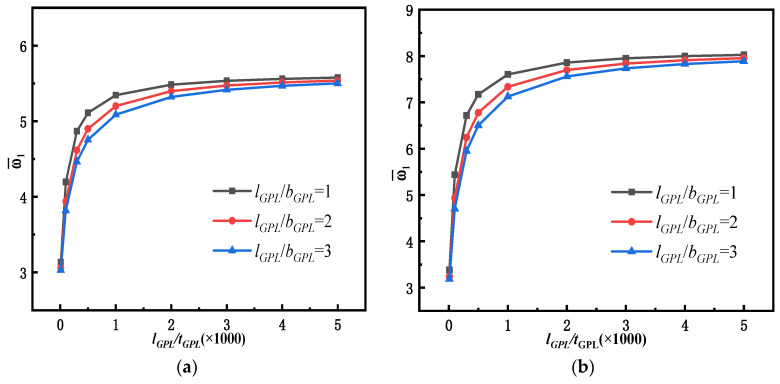

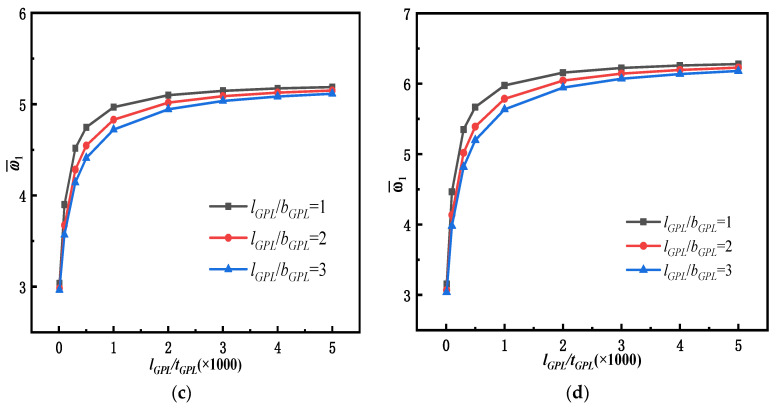
Variation of first-order dimensionless natural frequencies with respect to the length–thickness ratio (*l*/*h* = 20, $W_{GPL}^{t}=1\%$): (**a**) LDP, (**b**) PDPRS, (**c**)PDPRM, and (**d**) UDP.

Figure 10 shows the variation of the first-order dimensionless fundamental frequency of simply supported FG-GPLRC beams with varying sizes of GPLs. Figure 10 shows that the fundamental frequency of the beam increases substantially when the length–thickness ratio (*l_GPL_*/*t*_GPL_) of the GPLs increases to 1000 and then increases only slightly as the length–thickness ratio increases further. Whereas the total weight fraction of GPLs is kept constant, thinner GPLs result in a larger contact area between the GPLs and the matrix, so the GPLs are fully in contact with the matrix material, highlighting their high elastic modulus properties and considerably increasing the frequency of the beam accordingly. However, as GPLs become thinner, they are prone to curling behavior, changing from a planar configuration to a curved configuration, which cannot fully exploit their own properties and those of the composite material. Figure 10 also shows that under the four different distribution patterns of GPLs, the frequency of a square-GPL-enhanced FG beam is higher than that of a rectangular-GPL-enhanced FG beam because square GPLs have a larger specific surface area, resulting in superior performance enhancement.

### 5.3. Transient Response

**Figure 11:**
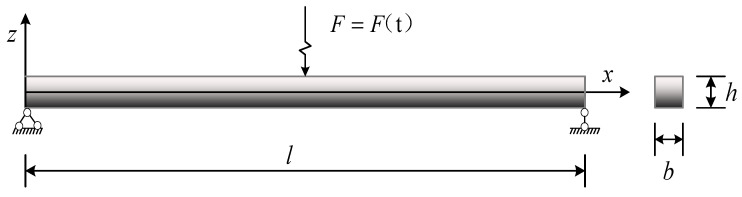
FG-GPLRC beam subjected to dynamic loads.

**Figure 12:**
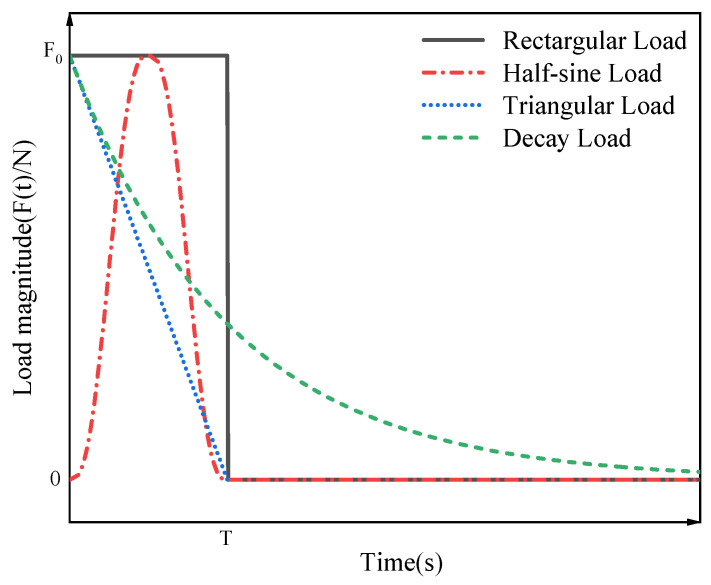
Four types of dynamic loads.

Figure 11 represents a simply supported FG-GPLRC beam with a rectangular cross section applied by a dynamic load over a period of time at mid span of the beam. Four dynamic load types are selected to investigate the effects of the graphene distribution pattern, mass, and size on the transient response, as shown in Figure 12, with a maximum value of dynamic load of *F*_0_ = 1 KN, *T* = 0.2 s. Fu and Chung [39] tested the loss tangent of epoxy resin under low-frequency external forces, with an obtained value of 0.030 ± 0.007. The same result was obtained by Fereidoon through experimental testing as 0.036 [40]. Thus, in the following numerical study, the low modal damping ratio (ζ) is assumed to be 0.03. The amplitude of the response to dynamic loads in the mid span of an FG-GPLRC beam is studied as an eigenvalue.

**Figure 13:**
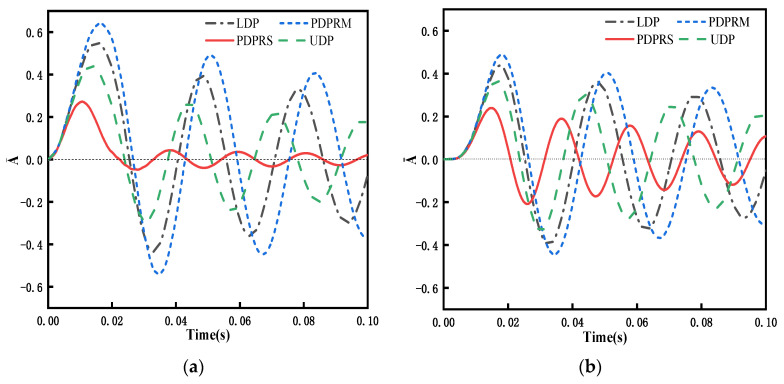

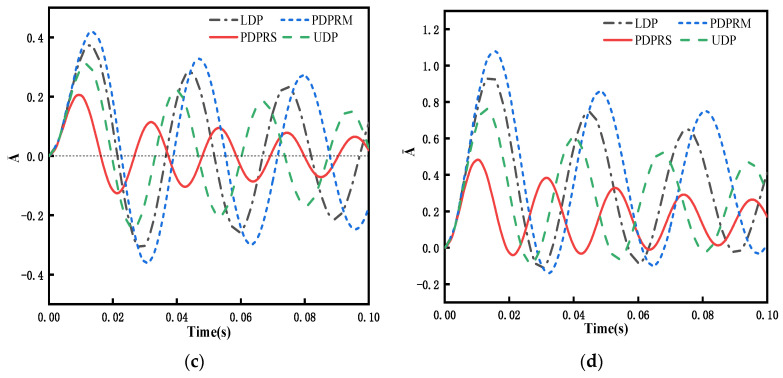
Dynamic response of an FG-GPLRC beam with four GPLs distribution patterns under four dynamic loads (*l*/*h* = 20, $W_{GPL}^{t}=1\%$): (**a**) rectangular load, (**b**) half-sine load, (**c**) triangular load, and (**d**) decay load.

Figure 13 compares the effects of four different GPL distribution patterns on the amplitude of the spanwise dynamic response of a simply supported FG-GPLRC beam under four dynamic loads. Due to the presence of damping in the system, the response amplitude becomes progressively smaller with time. Figure 13 shows that under the PDPRS of GPLs, the beam has the lowest dynamic response amplitude and the shortest response vibration period, whereas under the PDPRM of GPLs, the beam has the highest dynamic response amplitude and the longest response vibration period because PDPRS can distribute GPLs more on the upper and lower surfaces of the beam to effectively improve the flexural stiffness, which results in a higher vibration frequency and a shorter vibration period.

**Figure 14:**
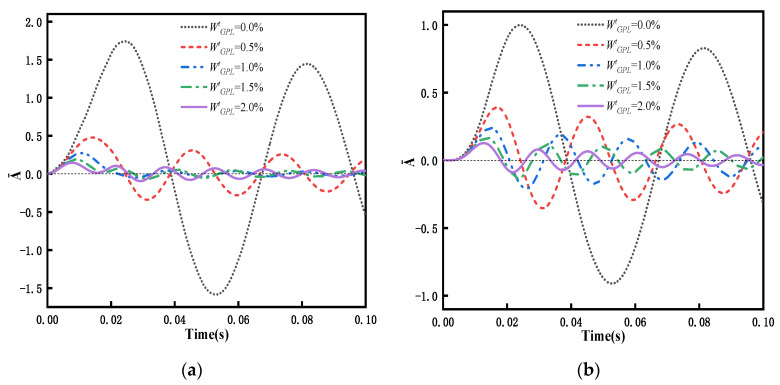

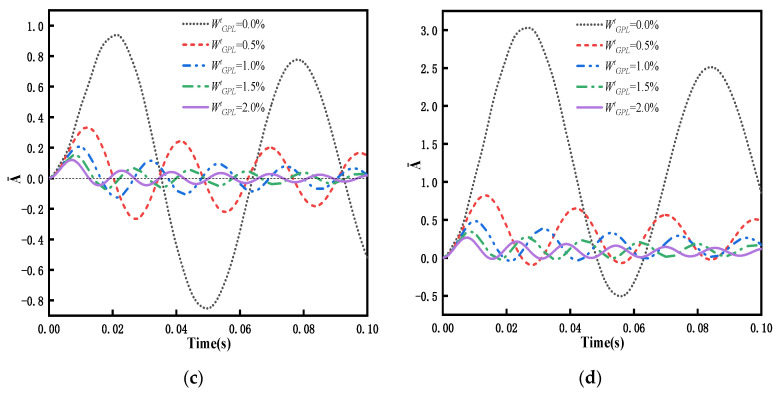
Dynamic response of a FG-GPLRC beam with PDPRS for various weight fraction of GPLs under four dynamic loads (*l*/*h* = 20): (**a**) rectangular load, (**b**) half-sine load, (**c**) triangular load, and (**d**) decay load.

In order to investigate the effects of total weight fraction of the GPLs on the dynamic response, a simply supported FG-GPLRC beam with the PDPRS of GPLs is considered under four kinds of dynamic load. The total weight fraction of the GPLs ranges from 0.0% to 2.0%, with an increment of 0.5%. Figure 14 represents the relationships between the dynamic responses and total weight fraction of the GPLs. Figure 14 shows that when $W_{GPL}^{t}$ = 0.0%, i.e., a pure epoxy beam, the amplitudes are largest, and the vibration periods are longest. With an increase in the total weight fraction of the GPL, the amplitudes of the dynamic responses decrease, and the vibration period gradually shortens. As described in Section 5.2, adding a small amount of GPLs can increase the flexural stiffness and the frequency of the beam, meaning that the amplitude of the dynamic response of the beam under dynamic load can be suppressed by adding a small amount of GPLs.

**Figure 15:**
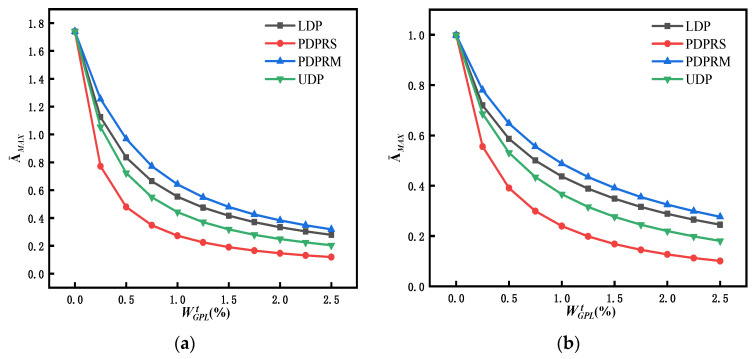

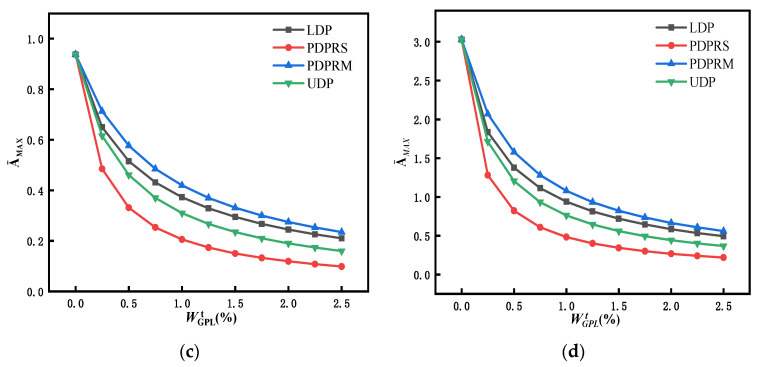
Variation in the maximum dimensionless amplitude at mid span of an FG-GPLRC beam with respect to the GPL weight fraction under four dynamic loads: (**a**) rectangular load, (**b**) half-sine load, (**c**) triangular load, and (**d**) decay load.

Consider dimensionless response vibration amplitude (${\bar{A}}_{MAX}$) at mid span of simply supported FG-GPLRC beams with different weight fractions and distribution patterns of GPLs. The total weight fraction of the GPLs ranges from 0.0% to 2.5%, with an increment of 0.25%. Figure 15 represents the relationships between ${\bar{A}}_{MAX}$ and $W_{GPL}^{t}$ under different distribution patterns of GPLs and different dynamic loads. The curves in Figure 15 clearly show that adding a small amount of GPLs can significantly reduce the maximum response amplitudes and enhance the bending performance of the beams. In the initial stage of the GPL weight fraction increase ($W_{GPL}^{t}$ from 0.0% to 1.0%), the amplitude (${\bar{A}}_{MAX}$) decreases obviously. As the GPL weight fraction continues to increase, the amplitude (${\bar{A}}_{MAX}$) continues to decrease, although not obviously. For the same GPL weight fraction, ${\bar{A}}_{MAX}$ of the beam with the PDPRS of GPLs decreases more than that of the other three distribution patterns (i.e., LDP, PDPRM, and UDP) under four kinds of dynamic load. It can be concluded that the PDPRS distributing more GPLs on the upper and lower surfaces of the beam is the most effective way to increase the stiffness of the beam, reduce the maximum response amplitude, and enhance the bending performance.

**Figure 16:**
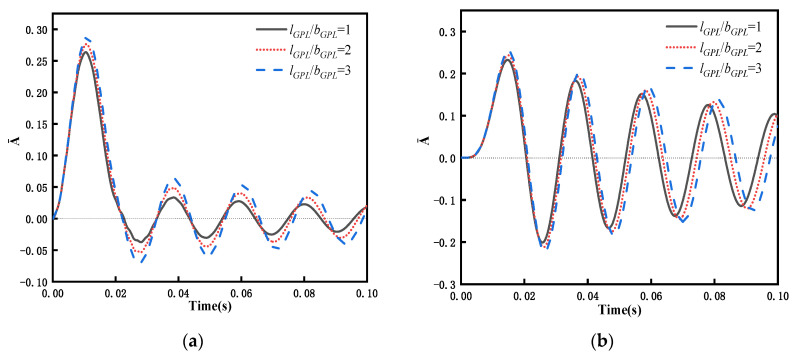

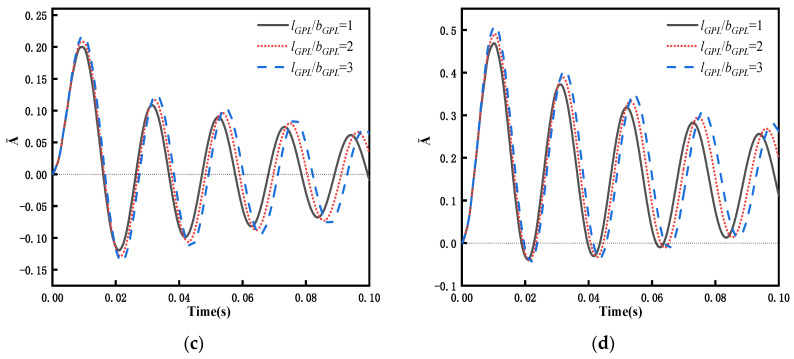
Dynamic response of an FG-GPLRC beam with PDPRS for various geometric shapes of GPLs under four dynamic loads (*l*/*h* = 20,$W_{GPL}^{t}=1\%$): (**a**) rectangular load, (**b**) half-sine load, (**c**) triangular load, and (**d**) decay load.

**Figure 17:**
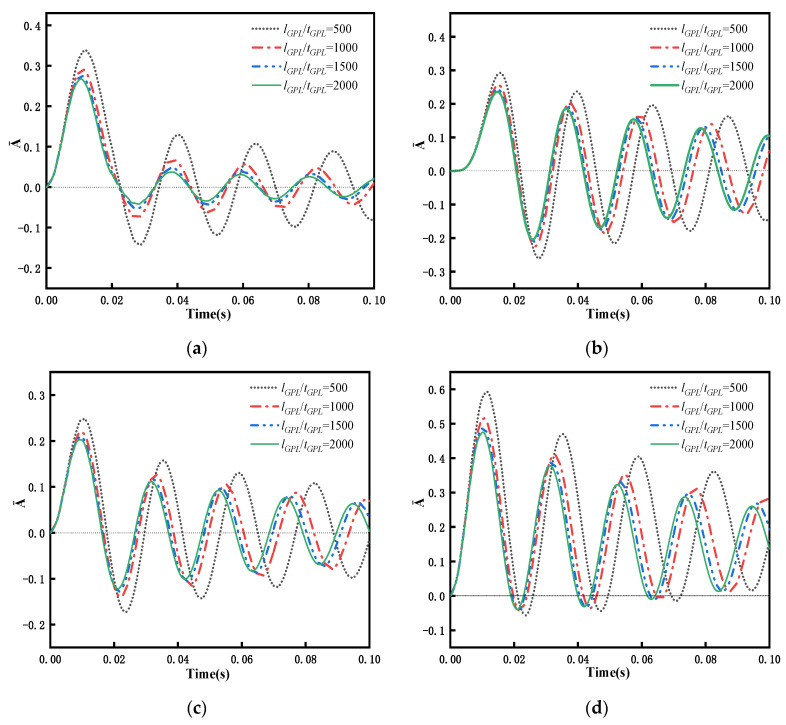
Dynamic response of an FG-GPLRC beam with PDPRS for various the length-to-thickness ratios of GPLs under four dynamic loads (*l*/*h* = 20, $W_{GPL}^{t}=1\%$): (**a**) rectangular load, (**b**) half-sine load, (**c**) triangular load, and (**d**) decay load.

Considering the PDPRS of GPLs achieves excellent performance with respect to transient response, the PDPRS is selected to study the effect of GPL size on transient response. An FG-GPLRC beam with a GPL weight fraction of $W_{GPL}^{t}$ = 1% is subjected to four different types of dynamic forces. Figure 16 shows the relationship between the dynamic response and the aspect ratio of GPLs. As shown in Figure 16, the response amplitude of the beam increases with increased aspect ratio, but the change in the response period is not obvious. The values of GPL length and width are taken from Table 2, i.e., *l_GPL_* × *b_GPL_* = 2.5 μm × 1.5 μm, and the thickness of GPLs is changed to further investigate the effect of thickness on the dynamic response of the beam. Figure 17 shows the relationship between the dynamic response and the length-to-thickness ratio of GPLs. As the GPLs become thinner, the dynamic response period of the beam becomes shorter, and the maximum amplitude becomes lower. The greatest decrease is observed under rectangular loading, when *l_GPL_*/*b_GPL_* changes from 500 to 2000, and the maximum amplitude decreases from 0.3384 to 0.2676—a decrease of 26%. This means that adding thinner GPLs can reduce the vibration amplitude of the beam structure under external action.

**Figure 18:**
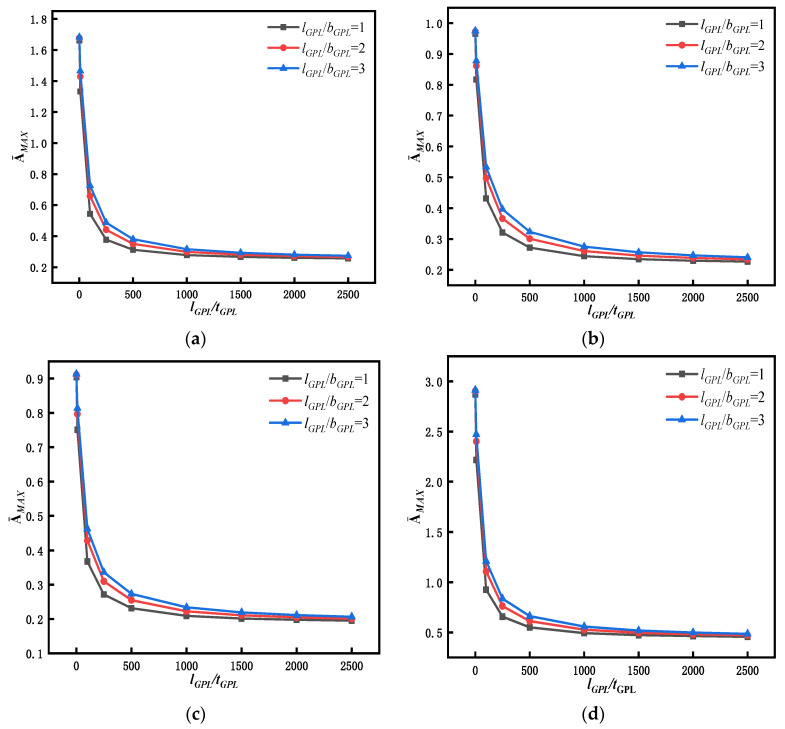
Variation in the maximum dimensionless amplitude at mid span of an FG-GPLRC beam with respect to the length-to-thickness ratio of GPLs under four dynamic loads (*l*/*h* = 20, C-C,$W_{GPL}^{t}=1\%$): (**a**) rectangular load, (**b**) half-sine load, (**c**) triangular load, and (**d**) decay load.

As a further illustration of the effects of GPL geometry on transient response, the changes in the aspect ratio (*l_GPL_*/*b_GPL_*) and the length–thickness ratio (*l_GPL_*/*t_GPL_*) are simultaneously considered. Figure 18 shows the variation curves of the maximum dimensionless response vibration amplitude (${\bar{A}}_{MAX}$) at mid span of the beam with different sizes and thicknesses of GPLs. The curves in Figure 18 show that in the process of increasing the length–thickness ratio to 500, ${\bar{A}}_{MAX}$ declines continuously, with a wide range of decline. However, as the length–thickness ratio exceeds 500 and increases further, the decline in ${\bar{A}}_{MAX}$ tends occurs gradually. When the *l_GPL_*/*t_GPL_* ratio is increased from 500 to 2000 for all operating conditions, the maximum dimensionless response amplitude decreases in the range of about 75% to 85%. When the total weight fraction of GPLs remains unchanged, relatively thin GPLs have a larger contact area between the GPLs and the matrix, which can transfer the load and stress better. When the GPLs become thinner and reach a certain thickness, they so thin that curling behavior occurs, and their advantages cannot be fully exploited to transfer the stress. Therefore, relatively thin GPLs can fully reduce the vibration amplitude of the beam, whereas excessively thin GPLs modulate the reduction effect to a certain extent.

Figure 18 also shows that the vibration amplitude of square-GPL-enhanced FG beams is lower than that of rectangular-GPL-enhanced beams for all four types of dynamic forces because square GPLs have a larger specific surface area, so they can transfer stress more effectively, resulting in an improved enhancement effect. This result provides a basis for future preparation of graphene-reinforced materials.

## 6. Conclusions

Free vibration characteristics and the dynamic behaviors of FG-GPLRC beams were investigated based on the finite element method. The GPL weight fraction varies continuously along the beam thickness according to a linear, parabolic, or uniform pattern. For GPL-reinforced composites, the effective Young’s modulus ($E_{C}$) is calculated according to the modified Halpin–Tsai micromechanics model, and the effective Poisson’s ratio ($\nu_{C}$) and mass density ($\rho_{C}$) are determined by the rule of mixture. Finite element formulations for the vibration analyses of FG-GPLRC beams are derived according to the principle of virtual work under the assumptions of the Euler–Bernoulli beam theory. A two-node beam element with six degrees of freedom is used to discretize the beam. Thus, the corresponding stiffness matrix and mass matrix, which contain information on the variation of material properties along the beam thickness, can be derived, and the equations of motion of the system are obtained for FG-GPLRC beams. On this basis, the natural frequencies of FG-GPLRC beams and the response amplitudes over time under the action of external forces are obtained using the FEM. The effects of four GPL distribution patterns, weight fractions, and dimensions of GPLs on the free vibration characteristics and transient response of the beams are investigated. Based on the numerical results, the following conclusions can be drawn:

The performance of the proposed FEM was assessed relative to some numerical results obtained by layering method and available in the published literature. Comparison results indicate that the proposed FEM is highly accuracy and efficiency and is therefore capable of analyzing FG-GPLRC beams.

Numerical results show that adding a small amount of GPLs can considerably increase the natural frequency of the beam and significantly reduce the dynamic amplitude of the beam under forced vibration. The maximum increase in the intrinsic frequency of beams is 170% when the weight fraction of GPLs is increased from 0% to 1%. Figure 14 shows that the dynamic amplitude of the beam under forced vibration can be reduced significantly as the weight fraction of GPLs is increased from 0% to 2%. Because graphene has very high Young’s modulus, adding a small amount of GPLs can considerably increase the stiffness of the FG-GPLRC beam and naturally improve its bending performance.

The distribution of GPLs in the beam has a considerable influence on the dynamic performance of the beam. Numerical results reveal that an FG-GPLRC beam with the PDPRS of GPLs has a higher natural frequency and lower dynamic amplitude than the other three distribution patterns, i.e., LDP, PDPRM, and UDP. Under the same GPL weight increment, such as a $W_{GPL}^{t}$ increase from 0.0% to 0.5%, the growth rate of the frequency is about 103% for the PDPRS of GPLs, 65% for the UDP of GPLs, 53% for the LDP of GPLs, and 43% for the PDPRM of GPLs. Figure 13 shows that under the PDPRS of GPLs, the beam has the lowest dynamic response amplitude, which indicates that distributing more GPLs on the upper and lower surfaces of the beam is the most effective method to increase the stiffness of the beam and improve its bending performance.

The geometrical size and shape of GPLs can affect the dynamic performance of the beam. Figure 10 indicates that under the condition a constant weight fraction, an FG-GPLRC beam with square GPLs and thinner GPLs has a higher natural frequency. Similarly, Figure 18 reveals that an FG-GPLRC beam with square GPLs and thinner GPLs less dynamic amplitude than a beam with rectangular GPLs and thicker GPLs. Because square GPLs and thinner GPLs have a larger specific surface areas, they can come into full contact with the matrix material and provide better load and stress transfer. Thus, for an FG-GPLRC beam, square GPLs and thinner GPLs have better potential to improve the flexural performance of the beam than rectangular GPLs and thicker GPLs.

The findings presented herein contribute to improved understanding of the influence of GPL weight fraction, distribution pattern, and dimensions on the mechanical properties of FG-GPLRC beams, which is beneficial with respect to the applications of FG-GPLRC beams in many fields, such as aerospace engineering, chemical engineering, civil engineering, etc. For example, the mechanical properties of beams play an important role in structural design and structural safety assessment. Thus, understanding how to add graphene to reinforced concrete beams for improved mechanical properties is helpful with respect to the application of research results the construction field.

## Figures and Tables

**Table 1:** Relationship between gradient factor $\lambda_{i}$ and $W_{GPL}^{t}$.

$\lambda_{1}$	$\lambda_{2}$	$\lambda_{3}$	$\lambda_{4}$	$W_{GPL}^{t}\;(\%)$
0	0	0	0	0
1	1.5	0.75	0.5	0.5
2	3	1.5	1	1
2x	3x	1.5x	x	x

**Table 2:** Material-related parameters.

*l_GPL_*/μm	*b_GPL_*/μm	*t_GPL_*/nm	*ν_M_*	*ν_GPL_*	*ρ_M_*/kg/m^3^	*ρ_GPL_*/kg/m^3^	*E_M_*/Gpa	*E_GPL_*/Gpa
2.5	1.5	1.5	0.34	0.186	1.2	1.06	3.0	1010

**Table 3:** Dimensionless fundamental frequencies of the FG-GPLRC beam with four GPL distribution patterns (*l*/*h* = 25, $W_{GPL}^{t}=1\%$).

Number of Elements	LDP	PDPRS	PDPRS	UDP
5	5.505	7.846	5.106	6.130
10	5.426	7.746	5.040	6.065
20	5.389	7.681	5.007	6.031
30	5.389	7.681	5.007	6.030
40	5.389	7.681	5.007	6.030
50	5.389	7.681	5.007	6.030
60	5.389	7.681	5.007	6.030

**Table 4:** Validation of the dimensionless fundamental frequencies of the FG-GPLRC beam.

Distribution Patterns	$W_{GPL}^{t}$	*l*/*h* = 20		*l*/*h* = 25			*l*/*h* = 100		
		Present	ABAQUS	Present	ABAQUS	Ref. [28]	Present	ABAQUS	Ref. [28]
LDP	0.0%	2.846	2.851	2.847	2.850	2.841	2.849	2.849	2.849
	0.5%	4.383	4.394	4.386	4.393	4.379	4.390	4.391	4.389
	1.0%	5.386	5.402	5.389	5.400	5.381	5.394	5.398	5.394
PDPRS	0.0%	2.846	2.851	2.847	2.850	2.841	2.849	2.849	2.849
	0.5%	5.789	5.756	5.791	5.765	5.764	5.794	5.788	5.793
	1.0%	7.679	7.609	7.681	7.629	7.637	7.686	7.681	7.683
PDPRM	0.0%	2.846	2.851	2.847	2.850	2.841	2.849	2.849	2.849
	0.5%	4.071	4.092	4.073	4.089	4.067	4.075	4.076	4.075
	1.0%	5.006	5.038	5.007	5.034	5.001	5.011	5.029	5.010
UDP	0.0%	2.846	2.851	2.847	2.850	2.841	2.849	2.849	2.849
	0.5%	4.714	4.721	4.715	4.720	4.705	4.718	4.719	4.718
	1.0%	6.029	6.040	6.030	6.038	6.019	6.036	6.036	6.035

**Table 5:** Comparisons of dimensionless natural frequencies of an FG-GPLRC beam (*l*/*h* = 20).

Distribution Pattern	Dimensionless Frequency	$W_{GPL}^{t}=0.0\%$	$W_{GPL}^{t}=0.5\%$		$W_{GPL}^{t}=1.0\%$	
		Value	Value	Growth Rate	Value	Growth Rate
LDP	${\bar{\omega }}_{1}$	2.846	4.383	54.006%	5.386	89.248%
	${\bar{\omega }}_{2}$	11.350	17.458	53.815%	21.438	88.881%
	${\bar{\omega }}_{3}$	25.408	38.907	53.129%	47.701	87.740%
PDPRS	${\bar{\omega }}_{1}$	2.846	5.789	103.408%	7.679	169.817%
	${\bar{\omega }}_{2}$	11.350	23.084	103.383%	30.620	169.780%
	${\bar{\omega }}_{3}$	25.408	51.677	103.389%	68.377	169.116%
PDPRM	${\bar{\omega }}_{1}$	2.846	4.071	43.043%	5.006	75.896%
	${\bar{\omega }}_{2}$	11.350	16.234	43.031%	19.962	75.877%
	${\bar{\omega }}_{3}$	25.408	36.343	43.038%	44.687	75.878%
UDP	${\bar{\omega }}_{1}$	2.846	4.714	65.636%	6.029	111.841%
	${\bar{\omega }}_{2}$	11.350	18.797	65.612%	24.044	111.841%
	${\bar{\omega }}_{3}$	25.408	42.079	65.613%	53.826	111.847%

(Growth rate = $\frac{{\bar{\omega }}_{GPLs}-{\bar{\omega }}_{empty}}{{\bar{\omega }}_{empty}}\times 100\%$).

## Data Availability

Not applicable.

## Appendix A

### Appendix A.1. The Explicit Forms of $C_{jl}$ and $C_{jw}$ (j = 1, 2, 3)

Distribution pattern of GPLs: LDP:

(A1) $$X_{1i}=\frac{\lambda_{1}\rho_{M}W_{GPL}^{0}(1-\eta_{i})+\rho_{GPL}(2-\lambda_{1}W_{GPL}^{0})}{\lambda_{1}\rho_{M}W_{GPL}^{0}(1-\xi_{i}\eta_{i})+\rho_{GPL}(2-\lambda_{1}W_{GPL}^{0})}$$

(A2) $$Y_{1i}=\frac{2\lambda_{1}W_{GPL}^{0}(\rho_{M}-\rho_{GPL}-\eta_{i}\rho_{M})}{h\left[\lambda_{1}W_{GPL}^{0}(\rho_{M}-\rho_{GPL}+\xi_{i}\eta_{i}\rho_{M})+2\rho_{GPL}\right]}$$

(A3) $$X_{2i}=\frac{h\left[\lambda_{1}W_{GPL}^{0}(\rho_{M}-\rho_{GPL}-\eta_{i}\rho_{M})+2\rho_{GPL}\right]}{2\lambda_{1}W_{GPL}^{0}(\rho_{M}-\rho_{GPL}+\xi_{i}\eta_{i}\rho_{M})}$$

(A4) $$Y_{2i}=\frac{\rho_{M}-\rho_{GPL}-\eta_{i}\rho_{M}}{\rho_{M}-\rho_{GPL}+\xi_{i}\eta_{i}\rho_{M}}$$

(A5) $$C_{1i}=\frac{1}{Y_{1i}}\ln\left|\frac{hY_{1i}+2X_{1i}}{-hY_{1i}+2X_{1i}}\right|+\frac{h}{Y_{2i}}-\frac{X_{2i}}{Y_{2i}^{2}}\ln\left|\frac{hY_{2i}+2X_{2i}}{-hY_{2i}+2X_{2i}}\right|$$

(A6) $$C_{2i}=\frac{h}{Y_{1i}}-\frac{X_{1i}}{Y_{1i}^{2}}ln\left|\frac{hY_{1i}+2X_{1i}}{-hY_{1i}+2X_{1i}}\right|-\frac{hX_{2i}}{Y_{2i}^{2}}+\frac{X_{2i}^{2}}{Y_{2i}^{3}}ln\left|\frac{hY_{2i}+2X_{2i}}{-hY_{2i}+2X_{2i}}\right|$$

(A7) $$C_{3i}=-\frac{hX_{1i}}{Y_{1i}^{2}}+\frac{X_{1i}^{2}}{Y_{1i}^{3}}ln\left|\frac{hY_{1i}+2X_{1i}}{-hY_{1i}+2X_{1i}}\right|+\frac{h^{3}}{12Y_{2i}}-\frac{hX_{2i}^{2}}{Y_{2i}^{3}}-\frac{X_{2i}^{3}}{Y_{2i}^{4}}ln\left|\frac{hY_{2i}+2X_{2i}}{-hY_{2i}+2X_{2i}}\right|$$

(i=l,w)

Distribution pattern of GPLs: PDPRS:

(A8) $$X_{4i}=\frac{h^{2}\rho_{GPL}}{4\lambda_{2}W_{GPL}^{0}(\rho_{M}-\rho_{GPL}+\xi_{i}\eta_{i}\rho_{M})}$$

(A9) $$Y_{3i}=\frac{\lambda_{2}W_{GPL}^{0}(\rho_{M}-\rho_{GPL}-\eta_{i}\rho_{M})}{h^{2}\rho_{M}}$$

(A10) $$Y_{4i}=\frac{\rho_{M}-\rho_{GPL}-\eta_{i}\rho_{M}}{\rho_{M}-\rho_{GPL}+\xi_{i}\eta_{i}\rho_{M}}$$

if $Y_{3i}>0$

(A11) $$C_{1i}=\frac{2}{\sqrt{Y_{3i}}}\arctan(\frac{h\sqrt{Y_{3i}}}{2})+\frac{h}{Y_{4i}}-2\sqrt{\frac{X_{4i}}{Y_{4i}^{3}}}\arctan(\frac{h\sqrt{Y_{4i}}}{2\sqrt{X_{4i}}})$$

(A12) $$C_{2i}=0$$

(A13) $$C_{3i}=\frac{h}{Y_{3i}}-\frac{2}{\sqrt{Y_{3i}^{3}}}\arctan(\frac{h\sqrt{Y_{3i}}}{2})+\frac{h^{3}}{12Y_{4i}}-\frac{hX_{4i}}{Y_{4i}^{2}}+2\sqrt{\frac{X_{4i}^{3}}{Y_{4i}^{5}}}\arctan(\frac{h\sqrt{Y_{4i}}}{2\sqrt{X_{4i}}})$$

if $Y_{3i}<0$

(A14) $$C_{1i}=\frac{1}{\sqrt{-Y_{3i}}}ln\left|\frac{h\sqrt{-Y_{3i}}+2}{h\sqrt{-Y_{3i}}-2}\right|+\frac{h}{Y_{4i}}+\sqrt{\frac{X_{4i}}{-Y_{4i}^{3}}}ln\left|\frac{h\sqrt{-Y_{4i}}+2\sqrt{X_{4i}}}{h\sqrt{-Y_{4i}}-2\sqrt{X_{4i}}}\right|$$

(A15) $$C_{2i}=0$$

(A16) $$C_{3i}=\frac{h}{Y_{3i}}-\frac{1}{\sqrt{-Y_{3i}^{3}}}ln\left|\frac{h\sqrt{-Y_{3i}}+2}{h\sqrt{-Y_{3i}}-2}\right|+\frac{h^{3}}{12Y_{4i}}+\frac{X_{4i}h}{Y_{4i}^{2}}+\sqrt{\frac{X_{4i}^{3}}{-Y_{4i}^{5}}}ln\left|\frac{h\sqrt{-Y_{4i}}+2\sqrt{X_{4i}}}{h\sqrt{-Y_{4i}}-2\sqrt{X_{4i}}}\right|$$

(i=l,w)

Distribution pattern of GPLs: PDPRM:

(A17) $$X_{5i}=\frac{\lambda_{3}W_{GPL}^{0}\left(\rho_{M}-\rho_{GPL}-\eta_{i}\rho_{M}\right)+\rho_{GPL}}{\lambda_{3}W_{GPL}^{0}(\rho_{M}-\rho_{GPL}+\xi_{i}\eta_{i}\rho_{M})+\rho_{GPL}}$$

(A18) $$Y_{5i}=\frac{4\lambda_{3}W_{GPL}^{0}(\rho_{GPL}-\rho_{M}+\eta_{i}\rho_{M})}{h^{2}\left[\lambda_{3}W_{GPL}^{0}\left(\rho_{M}-\rho_{GPL}+\xi_{i}\eta_{i}\rho_{M}\right)+\rho_{GPL}\right]}$$

(A19) $$X_{6i}=\frac{h^{2}\left[\lambda_{3}W_{GPL}^{0}\left(\rho_{M}-\rho_{GPL}-\eta_{i}\rho_{M}\right)+\rho_{GPL}\right]}{4\lambda_{3}W_{GPL}^{0}(\rho_{GPL}-\rho_{M}-\eta_{i}\rho_{M})}$$

(A20) $$Y_{6i}=\frac{\rho_{GPL}-\rho_{M}+\eta_{i}\rho_{M}}{\rho_{GPL}-\rho_{M}-\xi_{i}\eta_{i}\rho_{M}}$$

if $Y_{5i}>0$

(A21) $$C_{1i}=\frac{2}{\sqrt{Y_{5i}X_{5i}}}\arctan(\frac{h\sqrt{Y_{5i}}}{2\sqrt{X_{5i}}})+\frac{h}{Y_{6i}}+2\sqrt{\frac{X_{6i}}{Y_{6i}^{3}}}\arctan(\frac{h\sqrt{Y_{6i}}}{2\sqrt{X_{6i}}})$$

(A22) $$C_{2i}=0$$

(A23) $$C_{3i}=\frac{h}{Y_{5i}}-2\sqrt{\frac{X_{5i}}{Y_{5i}^{3}}}\arctan(\frac{h\sqrt{Y_{5i}}}{2\sqrt{X_{5i}}})+\frac{h^{3}}{12Y_{6i}}-\frac{hX_{6i}}{Y_{6i}^{2}}-2\sqrt{\frac{X_{6i}^{3}}{Y_{6i}^{5}}}\arctan(\frac{h\sqrt{Y_{6i}}}{2\sqrt{X_{6i}}})$$

if $Y_{5i}<0$

(A24) $$C_{1i}=\frac{1}{\sqrt{-Y_{5i}X_{5i}}}ln\left|\frac{h\sqrt{-Y_{5i}}+2\sqrt{X_{5i}}}{h\sqrt{-Y_{5i}}-2\sqrt{X_{5i}}}\right|+\frac{h}{Y_{6i}}-\sqrt{\frac{-X_{6i}}{Y_{6i}^{3}}}ln\left|\frac{h\sqrt{Y_{6i}}+2\sqrt{-X_{6i}}}{h\sqrt{Y_{6i}}-2\sqrt{-X_{6i}}}\right|$$

(A25) $$C_{2i}=0$$

(A26) $$C_{3i}=\frac{h}{Y_{5i}}+\sqrt{\frac{X_{5i}}{-Y_{5i}^{3}}}ln\left|\frac{h\sqrt{-Y_{5i}}+2\sqrt{X_{5i}}}{h\sqrt{-Y_{5i}}-2\sqrt{X_{5i}}}\right|+\frac{h^{3}}{12Y_{6i}}-\frac{X_{6i}h}{Y_{6i}^{2}}-\sqrt{\frac{-X_{6i}^{3}}{Y_{6i}^{5}}}ln\left|\frac{h\sqrt{Y_{6i}}+2\sqrt{-X_{6i}}}{h\sqrt{Y_{6i}}-2\sqrt{-X_{6i}}}\right|$$

(i=l,w)

Distribution pattern of GPLs: UDP:

(A27) $$C_{1i}=\frac{h\left[\lambda_{4}W_{GPL}^{0}\left(\rho_{M}-\rho_{GPL}+\xi_{i}\eta_{i}\rho_{M}\right)+\rho_{GPL}\right]}{\lambda_{4}W_{GPL}^{0}\left(\rho_{M}-\rho_{GPL}-\eta_{i}\rho_{M}\right)+\rho_{GPL}}$$

(A28) $$C_{2i}=0$$

(A29) $$C_{1i}=\frac{h^{3}\left[\lambda_{4}W_{GPL}^{0}\left(\rho_{M}-\rho_{GPL}+\xi_{i}\eta_{i}\rho_{M}\right)+\rho_{GPL}\right]}{12\left[\lambda_{4}W_{GPL}^{0}\left(\rho_{M}-\rho_{GPL}-\eta_{i}\rho_{M}\right)+\rho_{GPL}\right]}$$

(i=l,w)

### Appendix A.2. Explicit Forms of $C_{j}$ (j = 1, 2, 3)

Distribution pattern of GPLs: LDP:

(A30) $$L_{1}=\frac{\lambda_{1}W_{GPL}^{0}(\rho_{M}-\rho_{GPL})+2\rho_{GPL}}{\rho_{M}\lambda_{1}W_{GPL}^{0}}$$

(A31) $$J_{1}=\frac{2\rho_{M}-2\rho_{GPL}\lambda_{1}W_{GPL}^{0}}{h\rho_{M}\lambda_{1}W_{GPL}^{0}}$$

(A32) $$L_{2}=\frac{h\lambda_{1}W_{GPL}^{0}\left(\rho_{M}-\rho_{GPL}\right)+2h\rho_{GPL}}{2}$$

(A33) $$J_{2}=\rho_{M}-\rho_{GPL}\lambda_{1}W_{GPL}^{0}$$

(A34) $$C_{1}=\frac{1}{J_{1}}ln\left|\frac{hJ_{1}+2L_{1}}{-hJ_{1}+2L_{1}}\right|+\frac{h}{J_{2}}-\frac{L_{2}}{J_{2}^{2}}ln\left|\frac{hJ_{2}+2L_{2}}{-hJ_{2}+2L_{2}}\right|$$

(A35) $$C_{2}=\frac{h}{J_{1}}-\frac{L_{1}}{J_{1}^{2}}ln\left|\frac{hJ_{1}+2L_{1}}{-hJ_{1}+2L_{1}}\right|-\frac{hL_{2}}{J_{2}^{2}}+\frac{L_{2}^{2}}{J_{2}^{3}}ln\left|\frac{hJ_{2}+2L_{2}}{-hJ_{2}+2L_{2}}\right|$$

(A36) $$C_{3}=-\frac{hL_{1}}{J_{1}^{2}}+\frac{L_{1}^{2}}{J_{1}^{3}}ln\left|\frac{hJ_{1}+2L_{1}}{-hJ_{1}+2L_{1}}\right|+\frac{h^{3}}{12J_{2}}-\frac{hL_{2}^{2}}{J_{2}^{3}}-\frac{L_{2}^{3}}{J_{2}^{4}}ln\left|\frac{hJ_{2}+2L_{2}}{-hJ_{2}+2L_{2}}\right|$$

Distribution pattern of GPLs: PDPRS:

(A37) $$L_{3}=\frac{h^{2}\rho_{GPL}}{4\rho_{M}\lambda_{2}W_{GPL}^{0}}$$

(A38) $$J_{3}=\rho_{M}-\rho_{GPL}$$

(A39) $$C_{1}=\frac{h}{J_{3}}-2\sqrt{\frac{L_{3}}{J_{3}^{3}}}\arctan(\frac{h\sqrt{J_{3}}}{2\sqrt{L_{3}}})$$

(A40) $$C_{2}=0$$

(A41) $$C_{3}=\frac{h^{3}}{12J_{3}}-\frac{hL_{3}}{J_{3}^{2}}+2\sqrt{\frac{L_{3}^{3}}{J_{3}^{5}}}\arctan(\frac{h\sqrt{J_{3}}}{2\sqrt{L_{3}}})$$

Distribution pattern of GPLs: PDPRM:

(A42) $$L_{4}=\frac{\lambda_{3}W_{GPL}^{0}(\rho_{M}-\rho_{GPL})+\rho_{GPL}}{\rho_{M}\lambda_{3}W_{GPL}^{0}}$$

(A43) $$J_{4}=\frac{4\left(\rho_{GPL}-\rho_{M}\lambda_{3}W_{GPL}^{0}\right)}{h^{2}\rho_{M}\lambda_{3}W_{GPL}^{0}}$$

(A44) $$L_{5}=\frac{h^{2}\lambda_{3}W_{GPL}^{0}\left(\rho_{M}-\rho_{GPL}\right)+h^{2}\rho_{GPL}}{4\rho_{M}}$$

(A45) $$J_{2}=\rho_{GPL}-\rho_{M}\lambda_{3}W_{GPL}^{0}$$

(A46) $$C_{1}=\frac{2}{\sqrt{J_{4}L_{4}}}\arctan(\frac{h\sqrt{J_{4}}}{2\sqrt{L_{4}}})-\frac{h}{J_{5}}+2\sqrt{\frac{L_{5}}{J_{5}^{3}}}\arctan(\frac{h\sqrt{J_{5}}}{2\sqrt{L_{5}}})$$

(A47) $$C_{2}=0$$

(A48) $$C_{3}=\frac{h}{J_{4}}-2\sqrt{\frac{L_{4}}{J_{4}^{3}}}\arctan(\frac{h\sqrt{J_{4}}}{2\sqrt{L_{4}}})-\frac{h^{3}}{12J_{5}}+\frac{hL_{5}}{J_{5}^{2}}-2\sqrt{\frac{L_{5}^{3}}{J_{5}^{5}}}\arctan(\frac{h\sqrt{J_{5}}}{2\sqrt{L_{5}}})$$

Distribution pattern of GPLs: UDP:

(A49) $$C_{1}=\frac{h\rho_{M}\lambda_{4}W_{GPL}^{0}}{\lambda_{4}W_{GPL}^{0}\left(\rho_{M}-\rho_{GPL}\right)+\rho_{GPL}}$$

(A50) $$C_{2}=0$$

(A51) $$C_{3}=\frac{h^{3}\rho_{M}\lambda_{4}W_{GPL}^{0}}{12\left[\lambda_{4}W_{GPL}^{0}\left(\rho_{M}-\rho_{GPL}\right)+\rho_{GPL}\right]}$$
